# Targeting of Sna3p to the Endosomal Pathway Depends on Its Interaction with Rsp5p and Multivesicular Body Sorting on Its Ubiquitylation

**DOI:** 10.1111/j.1600-0854.2007.00610.x

**Published:** 2007-07-23

**Authors:** Marta Stawiecka-Mirota, Wojciech Pokrzywa, Joelle Morvan, Teresa Zoladek, Rosine Haguenauer-Tsapis, Danièle Urban-Grimal, Pierre Morsomme

**Affiliations:** 1 Institut Jacques Monod, CNRS, Universités Paris VI et VII, Département de Biologie Cellulaire 2 Place Jussieu 75005 Paris, France; 2 Department of Genetics, Institute of Biochemistry and Biophysics, Polish Academy of Sciences Pawinskiego 5a, 02-106 Warsaw, Poland; 3 Unité de Biochimie Physiologique, Institut des Sciences de la Vie, Université catholique de Louvain Croix du Sud 5-15, 1348 Louvain la Neuve, Belgium

**Keywords:** Lys63, multivesicular body, Nedd4, Rsp5, Sna3, ubiquitin, ubiquitin ligase, yeast

## Abstract

Rsp5p is an ubiquitin (Ub)-protein ligase of the Nedd4 family that carries WW domains involved in interaction with PPXY-containing proteins. It plays a key role at several stages of intracellular trafficking, such as Ub-mediated internalization of endocytic cargoes and Ub-mediated sorting of membrane proteins to internal vesicles of multivesicular bodies (MVBs), a process that is crucial for their subsequent targeting to the vacuolar lumen. Sna3p is a membrane protein previously described as an Ub-independent MVB cargo, but proteomic studies have since shown it to be an ubiquitylated protein. Sna3p carries a PPXY motif. We observed that this motif mediates its interaction with Rsp5p WW domains. Mutation of either the Sna3p PPXY motif or the Rsp5p WW3 domain or reduction in the amounts of Rsp5 results in the mistargeting of Sna3p to multiple mobile vesicles and prevents its sorting to the endosomal pathway. This sorting defect appears to occur prior to the defect displayed in *rsp5* mutants by other MVB cargoes, which are correctly sorted to the endosomal pathway but missorted to the vacuolar membrane instead of the vacuolar lumen. Sna3p is polyubiquitylated on one target lysine, and a mutant Sna3p lacking its target lysine displays defective MVB sorting. Sna3p undergoes Rsp5-dependent polyubiquitylation, with K63-linked Ub chains.

Ubiquitin (Ub) is conjugated to internal lysine residues of substrate proteins by a cooperative set of enzymes known as E1 Ub-activating enzyme, E2 Ub-conjugating enzymes and E3 Ub-protein ligases, these latter being responsible for substrate recognition. In addition to its function in proteasome-mediated degradation, Ub has been described to play a crucial role during several stages of intracellular trafficking. Covalent modification by Ub acts as a signal for internalization of most, or perhaps even all, yeast plasma membrane proteins and a number of receptors/channels/transporters in mammals (1). Ubiquitin is also required for sorting of membrane proteins to internal vesicles, which invaginate, budding into the lumen of late endosomes, resulting in the formation of multivesicular bodies (MVBs) (2). These proteins are then delivered to the vacuolar lumen after fusion of MVBs with the vacuole/lysosome. Multivesicular body sorting involves a machinery conserved from yeast to humans, composed of several endosomal sorting complexes required for transport (ESCRTs), some of which contain a subunit carrying an Ub-binding domain (3). These complexes are thought to act sequentially in recognition of ubiquitylated cargoes. Finally, a few cases have been reported in which covalent modification by Ub appears crucial for Golgi exit of yeast transporters sorted to the endosomal pathway for vacuolar degradation instead of plasma membrane targeting (4–6).

In yeast, a key player that is active during these three Ub-dependent trafficking steps is the E3 Rsp5p. Rsp5p is the only member in yeast of the Nedd4 subfamily of E3 ligases and is essential for cell viability. All the family members harbor a C2 domain (binding of phospholipids and/or proteins), followed by two to four WW domains in their N-terminal regions and a C-terminal catalytic HECT (homologous to E6-AP COOH terminus) domain. Rsp5p-dependent, Ub-dependent MVB sorting has been analyzed with precision for only a few cargoes, for example the carboxypeptidase Cps1p and the polyphosphate phosphatase Phm5p (7–9). Several cases of MVB cargoes that apparently undergo Ub-independent MVB sorting have also been reported. This was first described for the yeast Sna3p (10), and other examples have also been reported in mammals (11,12).

Sna3p is a small protein of unknown function possessing two transmembrane domains and ultimately targeted in the vacuolar lumen. It belongs to a small family of conserved proteins present in plant and fungi. The budding yeast has four Sna proteins (Sna1–4) that have different localizations in the cell. Sna1p is localized at the plasma membrane and involved in high tolerance to salt stress (13). Sna3p is targeted to the vacuolar lumen by the endosomal pathway. Two observations argue in favor of the hypothesis that Sna3p might undergo Ub-independent MVB sorting (10). First, Sna3-green fluorescent protein (GFP) is still correctly sorted to internal MVB vesicles under conditions of Ub depletion, which impairs MVB sorting of certain other cargoes. Second, a mutant form of Sna3-GFP lacking two lysines (K19 and 125) – the only potential Sna3 cytoplasmic lysines – is still correctly targeted to the vacuolar lumen. It has thus been postulated that ubiquitylation marks some, but not all, membrane proteins for sorting into the interior of the vacuole. Surprisingly, two proteomic studies reported that Sna3p is ubiquitylated (target K125) (14,15).

In the present paper, we present a further characterization of the Golgi to vacuole trafficking of Sna3p together with its ubiquitylation status. We focus on the potential role of Rsp5p in the ubiquitylation and trafficking of Sna3p. Our data lead to conclusions that differ significantly from those presented in three other papers published while our work was under revision (16–18).

## Results

### Sna3p physically interacts with Rsp5p via its PY motif

**Figure 1:**
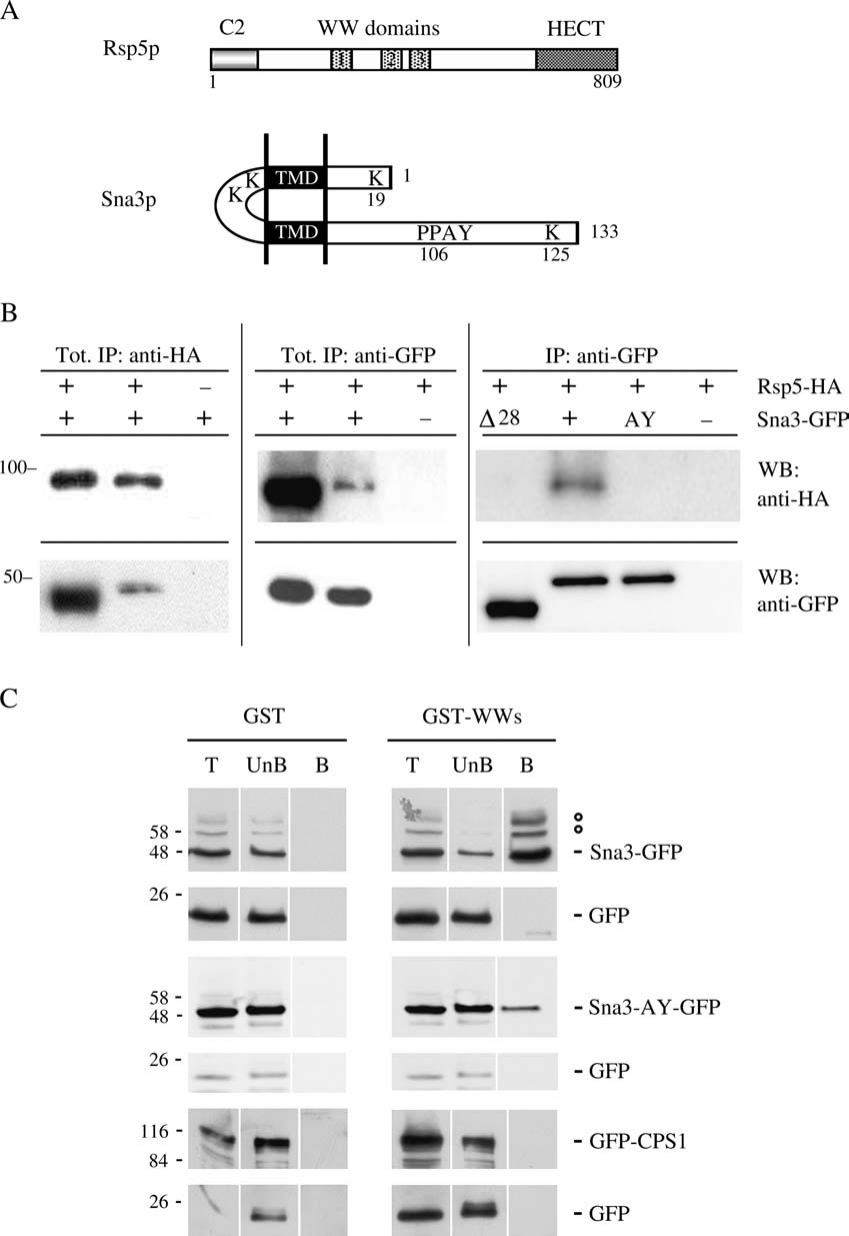
**Binding of the PY motif of Sna3p to the WW domains of Rsp5p.**A) Schematic representation of Rsp5p and Sna3p. B) Immunoprecipitation was performed with 170 μg of total extracts from cells producing HA-tagged Rsp5p under the control of its own promoter and transformed by a plasmid encoding GFP-tagged Sna3p wild type or mutants under the control of *TPI1* promoter. Tagged proteins were immunoprecipitated with anti-HA or anti-GFP antibodies. Cells producing untagged Rsp5p or Sna3p were used as controls. Immunoprecipitated proteins were detected by immunoblotting with anti-HA and anti-GFP antibodies. Tot: 6% of the solubilized proteins were loaded on the gel. IP: 50% of the immunoprecipitated material was loaded on the gel. C) Glutathione S-transferase and GST-WW2/WW3 Rsp5p domain recombinant proteins bound to glutathione–Sepharose beads were incubated with extracts from cells producing Sna3-GFP, Sna3-AY-GFP or GFP-Cps1p. Total extract (T), unbound (UnB) and bound (B) fractions were analyzed by Western blotting with anti-GFP. °: These bands were demonstrated to be ubiquitylated Sna3p species (see later). IP: immunoprecipitation, WB: western-blot.

Rsp5p is a modular protein with an N-terminal C2 domain that may interact with membranes (by binding to lipids or membrane proteins), three tryptophan-rich WW domains involved in protein–protein interactions with proline-rich peptides and a C-terminal catalytic HECT domain (Figure 1A). There is now a considerable body of data to suggest that the tryptophan-rich WW domains of the Nedd4 family members, or a subset of these domains, are involved, either directly or indirectly, in substrate recognition. Based on the presence of signature residues, a classification scheme has been proposed for WW domains (19). The three WW domains of Rsp5p belong to the group I domains that mediate protein–protein interactions via the PPXY (PY) motifs (20). The biosynthetic vacuolar protein Sna3p possesses a PY motif (sequence PPAY) in its C-terminus cytoplasmic part (Figure 1A). This prompted us to investigate the possibility that Sna3p interacts with the Ub ligase Rsp5p via its PPAY sequence. We conducted co-immunoprecipitation experiments with cells expressing both influenza hemagglutinin protein (HA)-tagged Rsp5p under the control of its own promoter and GFP-tagged Sna3p under the control of the *TPI1* promoter (Figure 1B). First, we performed immunoprecipitations with antibodies recognizing HA and then probed immunoprecipitated proteins with HA or GFP antibodies. Figure 1B shows that Sna3-GFP was efficiently co-immunoprecipitated with HA-Rsp5p. Fifteen percent of the solubilized HA-Rsp5p was recovered after immunoprecipitation, and 4% of the solubilized Sna3-GFP was co-immunoprecipitated. In contrast, Sna3-GFP was not detected in the control experiment, which was performed on cells producing untagged Rsp5p. Immunoprecipitations were also performed with antibodies recognizing GFP. Immunoprecipitated proteins were probed with HA or GFP antibodies revealing the presence of both Rsp5p-HA and Sna3p-GFP after immunoprecipitation. Fifteen percent of the solubilized HA-Rsp5p was recovered after immunoprecipitation, and 4% of the solubilized Sna3-GFP was co-immunoprecipitated. Rsp5p-HA was not detected in the control experiment, which was performed on cells expressing untagged Sna3p. These results indicate that Sna3p interacts either directly or indirectly with Rsp5p. To determine whether the Sna3p PPAY sequence is required for Rsp5p binding, we performed co-immunoprecipitation assays from HA-tagged Rsp5p-expressing cells transformed by a plasmid encoding a mutant form of Sna3-GFP in which the PY motif was deleted (mutant Δ28) or modified for AAAY (mutant AY). As shown in Figure 1B, these two variant forms that lack the PY motif were unable to interact with Rsp5p-HA, confirming the importance of the PY motif for this interaction and supporting the idea of a direct interaction.

Next, to investigate whether Sna3p specifically interacted with the WW domains of Rsp5p, we used a glutathione S-transferase (GST) fusion protein expressing the WW2/WW3 domains of Rsp5p (21). These domains have been specifically shown to be involved in the interaction of Rsp5p with several proteins that possess a PY motif (20,22,23). Using purified recombinant GST-WW2/WW3 Rsp5p domains, we performed a pull-down assay with Sna3-GFP-expressing cell lysate. Lysates from cells expressing an HA-tagged version of the Rsp5p partner Bul1p possessing a PY motif (24) or a GFP-tagged version Cps1p, a vacuolar enzyme lacking a PY motif but ubiquitylated by Rsp5p (8,9), were used as positive and negative controls, respectively. When using the same binding conditions, we observed a specific and strong binding of Bul1p to GST-WW2/WW3 but no binding to the GST control (not shown), and the same held true for Sna3p (Figure 1C). Binding decreased in the case of the mutant Sna3-AY (or Sna3-Δ28, not shown), and the control Cps1p did not bind at all to the GST-WW2/WW3. We conclude from these *in vitro* association assays that the WW2/WW3 domains of Rsp5p are responsible for its interaction with Sna3p, which is likely direct.

### Interaction with Rsp5p is required for sorting of Sna3p to the endosomal pathway

**Figure 2:**
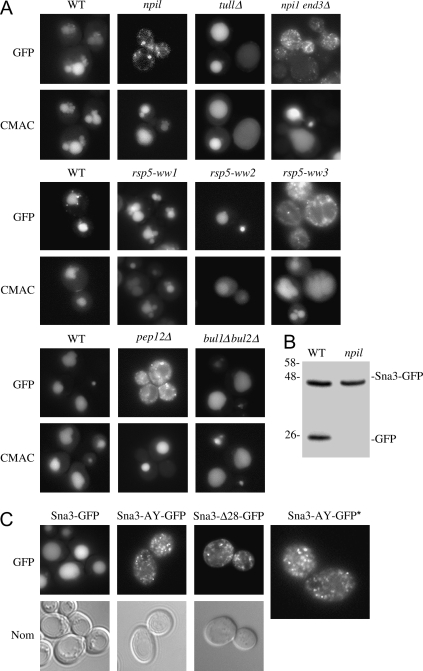
**Targeting of Sna3-GFP to the vacuolar lumen is abolished in *rsp5* mutant cells and if the Sna3p PY motif is altered.**Microscopic images of Sna3-GFP in living cells. Cells transformed by a Sna3-GFP plasmid under the control of the *TPI1* promoter were grown to mid-exponential growth phase and the fluorescence was examined under the microscope. A) Green fluorescent protein and CMAC staining in 27061b parental strain and its derivatives, *npi1*, *tul1Δ* and *npi1 end3Δ* mutant cells; in the BG1 parental strain and derivative cells deleted for *RSP5* and producing a centromeric plasmid version of Rsp5p wild type (WT) or mutated in the WW1, WW2 or WW3 domain; and in BY parental strain, *pep12Δ* and *bul1*Δ*bul2*Δ mutant cells. The identity of the vacuole was confirmed by staining with the dye CMAC as described in *Materials and Methods*. B) Western blots from total protein extracts from WT and *npi1* mutant cells producing Sna3-GFP were probed with anti-GFP antibodies. The sizes of molecular weight markers are indicated. C) Cells transformed by a plasmid encoding GFP-tagged WT or Sna3p variants were grown to mid-exponential phase and cells were examined for fluorescence and with Nomarski (Nom) optics. *, the image of Sna3-AY-GFP is magnified.

**Figure 3:**
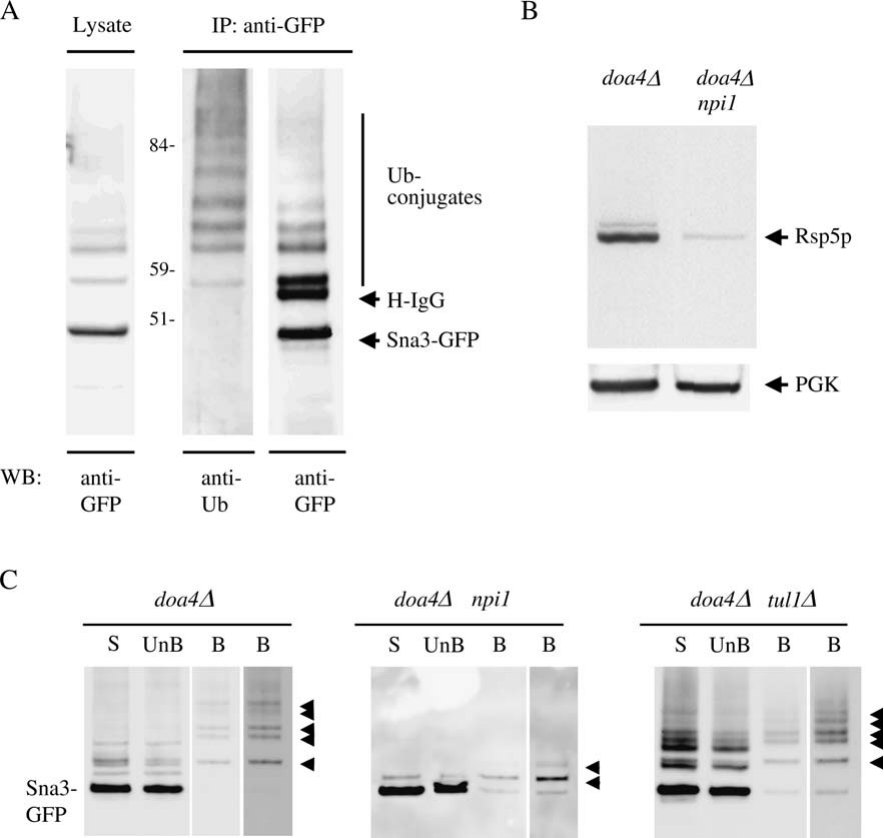
**Sna3p is ubiquitylated by the E3 ligase Rsp5p.**A) Green fluorescent protein-tagged version of Sna3p was detected either by immunoblotting of a cell lysate from wild-type (WT) cells with anti-GFP or by immunoblotting with anti-Ub or anti-GFP after immunoprecipitation with anti-GFP. Molecular weight markers sizes are indicated. B) Rsp5p and PGK, a loading control protein, were detected by immunoblotting of a cell lysate from *doa4Δ* and *doa4Δnpi1* cells with anti-mNedd4 and anti-PGK antibodies, respectively. C) *doa4Δ*, *doa4Δnpi1* and *doa4Δtul1Δ* cells co-producing Sna3-GFP and *CUP1* promoter-driven 6His-Ub were grown to mid-exponential phase. CuSO_4_ (100 μm) was added, and the cells were incubated for an additional 3 h to induce the *CUP1* promoter. Extracts of 6 × 10^8^ to 7 × 10^8^ cells were fractionated as described in *Materials and Methods*. All experiments were conducted in identical conditions of growth and cell fractionation in at least three independent experiments. Solubilized membranes were passed through Ni-NTA columns. The unbound fractions were collected and the 6His-tagged ubiquitylated proteins were then eluted using a buffer containing 200 mm imidazole. Aliquots of each fraction were resolved by electrophoresis and analyzed by Western immunoblotting with anti-GFP antibodies. Solubilized membrane fractions (S), unbound fraction (UnB) and purified 6His-tagged proteins (B) are shown. The lane on the right is overexposed. Arrowheads indicate Sna3-GFP conjugated to 6His-tagged Ub moieties. IP: immunoprecipitation, WB: western-blot.

We then investigated the potential role of this Rsp5p interaction in the sorting of Sna3p by analyzing the subcellular distribution of Sna3p fused to the GFP in various *rsp5* mutant cells relative to wild-type cells. When expressed in wild-type cells of various genetic backgrounds, Sna3-GFP was delivered to the lumen of the vacuole, resulting in GFP fluorescence in the vacuole interior, as shown by the identical localization obtained with the vacuolar cell tracker blue CMAC dye (Figure 2A). We then investigated the role played by Rsp5p in the delivery of Sna3-GFP to the vacuole (Figure 2A). We first used viable *npi1* mutant cells, *npi1* being a mutant allele of *RSP5* with a reduced expression of Rsp5p because of the insertion of a Ty element in the *RSP5* promoter (25,26). Using antibodies against mNedd4, the mouse homologue of Rsp5p, we were able to confirm that this mutant produced very small amounts of Rsp5p (Figure 3B). In *npi1* mutant cells, Sna3-GFP accumulated in very small and mobile structures, probably vesicles (Figure 2A). These diffuse stained dots were also observed in temperature-sensitive *rsp5-101*ts mutant cells shifted to nonpermissive temperature (data not shown). Sna3-GFP delocalization was identical in *npi1 end3Δ* cells, also defective for the internalization step of endocytosis, indicating that Sna3-GFP was sorted directly from the Golgi to these small vesicles rather than via the plasma membrane followed by subsequent endocytosis. This vesicular pattern is similar to that observed upon expression of Sna3-GFP in *pep12Δ*, defective for the fusion of vesicles with the late endosome.

We investigated the role of each of the WW domains of Rsp5p, using *rsp5*-null cells expressing centromeric plasmids carrying either wild-type *RSP5* or WW mutant *RSP5*. The *rsp5-w1/2/3* mutant alleles correspond to individual mutations of the WW domains that abolish the interaction of these WW domains with other proteins by altering two conserved amino acids (27). Strikingly, Sna3-GFP accumulated in the same mobile vesicles only in cells expressing *rsp5-w3* (Figure 2A). This was not the case in cells expressing *rsp5-w1* or *rsp5-w2* in which Sna3-GFP was correctly sorted to the vacuolar lumen. Deletion of the C2 domain of Rsp5p had no effect on the sorting of Sna3p (data not shown). Interestingly, the sorting defect of Sna3p in *rsp5* mutants differs from those observed for several other MVB cargoes. Other groups and we previously showed that GFP-Cps1p and GFP-Phm5p, two other biosynthetic vacuolar enzymes, were correctly sorted to the endosomal pathway but missorted to the limiting membrane of the vacuole instead of to the vacuolar lumen in cells lacking sufficient Rsp5p activity, including the *npi1* mutant cells (7–9). The Sna3p sorting defect we observed in all the above *rsp5* mutants was clearly different and most probably occurred before MVB sorting. Thus, beyond its role in the ubiquitylation of certain biosynthetic cargoes for their correct sorting into the MVBs, Rsp5p may be required for the delivery to the late endosomal compartment of vesicles emanating from the Golgi. The third WW domain of Rsp5p is required for this sorting event. The E3 ligase Tul1p, previously shown to be essential for sorting of GFP-Cps1p and GFP-Phm5 into MVBs (9,28), had no effect on the sorting of Sna3-GFP (Figure 2A). Similarly, Sna3-GFP sorting was normal in cells lacking the PY-containing Bul1/2 adaptors, required for Rsp5-dependent Golgi exit of several yeast transporters (5,6).

As we had established that the PY motif of Sna3p is required for the interaction with WW domains of Rsp5p, we investigated whether this motif was also involved in Sna3p trafficking. We introduced deletions in the cytoplasmic C-terminus part of Sna3p containing the PY motif and investigated the localization of the corresponding variant proteins fused to GFP (Figure 2C). Sna3-GFP lacking the last 28 C-terminal amino acids including the PY motif did not reach the vacuolar lumen and instead localized to small mobile dots. In contrast, the Sna3p mutant, deleted for the 24 terminal residues and still carrying the PY motif, was correctly located to the vacuolar lumen (data not shown). This difference shows that the PY motif plays a key role in Sna3p sorting. Indeed, when the PPAY sequence was changed to AAAY, Sna3-GFP also localized in mobile dots. These results confirm the dependency of the Sna3p localization on the PPAY motif and on its interaction with the WW domains of Rsp5p.

To confirm our fluorescence data for Sna3p localization in a more quantitative way, we performed Western blot analysis of Sna3-GFP present in total extracts of wild-type and *npi1* cells. In wild-type cells, entry into the vacuole exposed the GFP tag to vacuolar proteases, resulting in the cleavage of GFP and the appearance of a signal corresponding to the free GFP (Figure 2B). This band was not visible in *npi1* cell extracts, confirming that the GFP was not exposed to vacuolar proteases and did not reach the MVB internal vesicles and the vacuolar lumen.

### Sna3p is modified with the Ub moieties by the Ub ligase Rsp5p

On a Western blot probed with anti-GFP antibodies, the Sna3-GFP profile from wild-type cell lysate displayed, above the main Sna3-GFP band, a pattern of several bands with lower mobility. These low-mobility species form a ‘ladder’ of Ub-conjugated bands with successive additions of 7.6 kDa to the main signal (Figure 3A). Immunoprecipitations were performed on the corresponding lysate with antibodies recognizing GFP, and immunoprecipitated proteins were probed with anti-Ub or anti-GFP antibodies. Probing with anti-Ub revealed a ladder of more than nine bands corresponding to Sna3-GFP conjugated to Ub. Some Ub-conjugated bands were also revealed, although to a lesser extent, by probing the Western blot with anti-GFP. It should be noted that the band corresponding to the addition of the first Ub (monoubiquitylation) was more apparent with anti-GFP than with anti-Ub antibodies.

As Rsp5p, together with the RING-finger E3 Tul1p, ubiquitylates Cps1p for its correct sorting to the MVB (8,9,28), we analyzed the potential role played by Rsp5p and Tul1p in the ubiquitylation of Sna3-GFP. We improved the level of detection of Ub–Sna3p conjugates by using cells lacking the Doa4p Ub isopeptidase, which is involved in the deubiquitylation of endocytic and biosynthetic vacuolar cargoes (29). A lack of Doa4p function also results in the depletion of free Ub, interfering with many ubiquitylation processes including the selective sorting of Ub-tagged MVB cargoes (10,30). Because it is possible to complement *doa4Δ* cells with a plasmid-encoded 6His-Ub, we were able to check the ubiquitylation pattern of Sna3p in cells mutated for Rsp5p and deleted for Tul1p. We produced 6His-tagged Ub from a multicopy plasmid in *doa4Δ*, *doa4Δnpi1* and *doa4Δtul1Δ* cells to allow detection of ubiquitylated proteins specifically based on their acquisition of the 6His tag. We first checked that the overexpression of 6His-tagged Ub did indeed result in a high tagged to untagged ratio of Ub in *doa4Δ* cells defective for *TUL1* and *RSP5/NPI1* and producing 6His-tagged Ub under the control of the *CUP1* promoter [data not shown and (9)].

Cells were grown to mid-exponential phase and 6His-tagged Ub production was induced by adding copper for 3 h before harvest. After cell lysis, protein extracts corresponding to equivalent numbers of mutant cells were subjected to cell fractionation. The membrane fractions were solubilized and loaded onto a Ni-NTA Superflow resin. Aliquots of the solubilized pellet (S) from *doa4Δ* cells producing 6His-tagged Ub cells, together with the corresponding aliquots of bound (B) and unbound material (UnB), were resolved by electrophoresis and subjected to Western blotting and then the proteins of interest were detected using anti-GFP antibodies (Figure 3C). The electrophoretic pattern obtained with the solubilized P13 pellet was similar to that obtained with total proteins from lysate. Ubiquitylated bands were specifically retained on nickel columns. Above a faint band that corresponded to the residual retention of the core Sna3-GFP, more than six more slowly migrating bands were specifically retained, which corresponded to Sna3-GFP modified with Ub moieties in *doa4Δ* cells. The mobility of these bands was consistent with the hypothesis that the lower band has a single added Ub and the upper bands have increasing numbers of added Ub moieties. Aliquots of bound and unbound fractions corresponding to equivalent amounts of solubilized membrane from *doa4Δtul1Δ* cells exhibited the Ub profile the same as that observed with *doa4Δ* cells, indicating that Tul1p plays no role in Sna3-GFP ubiquitylation. In contrast, *doa4Δnpi1* cells had a large deficit in Ub conjugates. The ladder of multi-Ub was not detectable and was replaced by a strong band corresponding to monoubiquitylation and a faint band of lower mobility that might correspond to di-Ub. A lysate from *doa4Δnpi1 tul1Δ* triple-mutant cells exhibited the Ub pattern the same as that of a *doa4Δnpi1* lysate (data not shown). A second approach was used to determine the ubiquitylated profile of Sna3-GFP in *rsp5*ts mutant cells. Sna3-GFP was induced from a *GAL* promoter in parental and *rsp5-101*ts cells shifted to 37°C for 1 h prior to galactose induction. Sna3-GFP was then immunoprecipitated from the corresponding lysates and revealed by Western blotting with anti-Ub antibodies. Sna3-GFP was again essentially found to be modified with one added Ub and, to a lesser extent, a second Ub moiety in *rsp5*ts mutant cells (data not shown).

Our results all demonstrate that Sna3p is polyubiquitylated by Rsp5p. Whether the residual Rsp5p activity present in the two types of *rsp5* mutants used in this study or another E3 ligase activity is responsible for the monoubiquitylation of Sna3-GFP, at least in the absence of a normal amount of Rsp5p, remains to be tested. We show here that the RING-finger E3 Tul1p does not take part in this process.

### Sna3p is polyubiquitylated on one target lysine, Lys125

Ubiquitylation has been demonstrated to be a key event in the sorting of membrane proteins first to MVBs and then to the vacuolar lumen. We show here that Sna3-GFP is ubiquitylated. Moreover, two different proteomic approaches have recently shown that Sna3p is ubiquitylated in at least one of the four lysine residues present in the protein, Lys125 (14,15). In contrast, it has previously been shown that the Sna3-K19,125R–GFP fusion protein, in which the two lysine residues (K19 and 125) in the predicted cytoplasmic domain of Sna3p were replaced by Arg, was still targeted to the vacuolar lumen (10). The authors suggested that Ub is not required for Sna3p vacuolar sorting.

**Figure 4:**
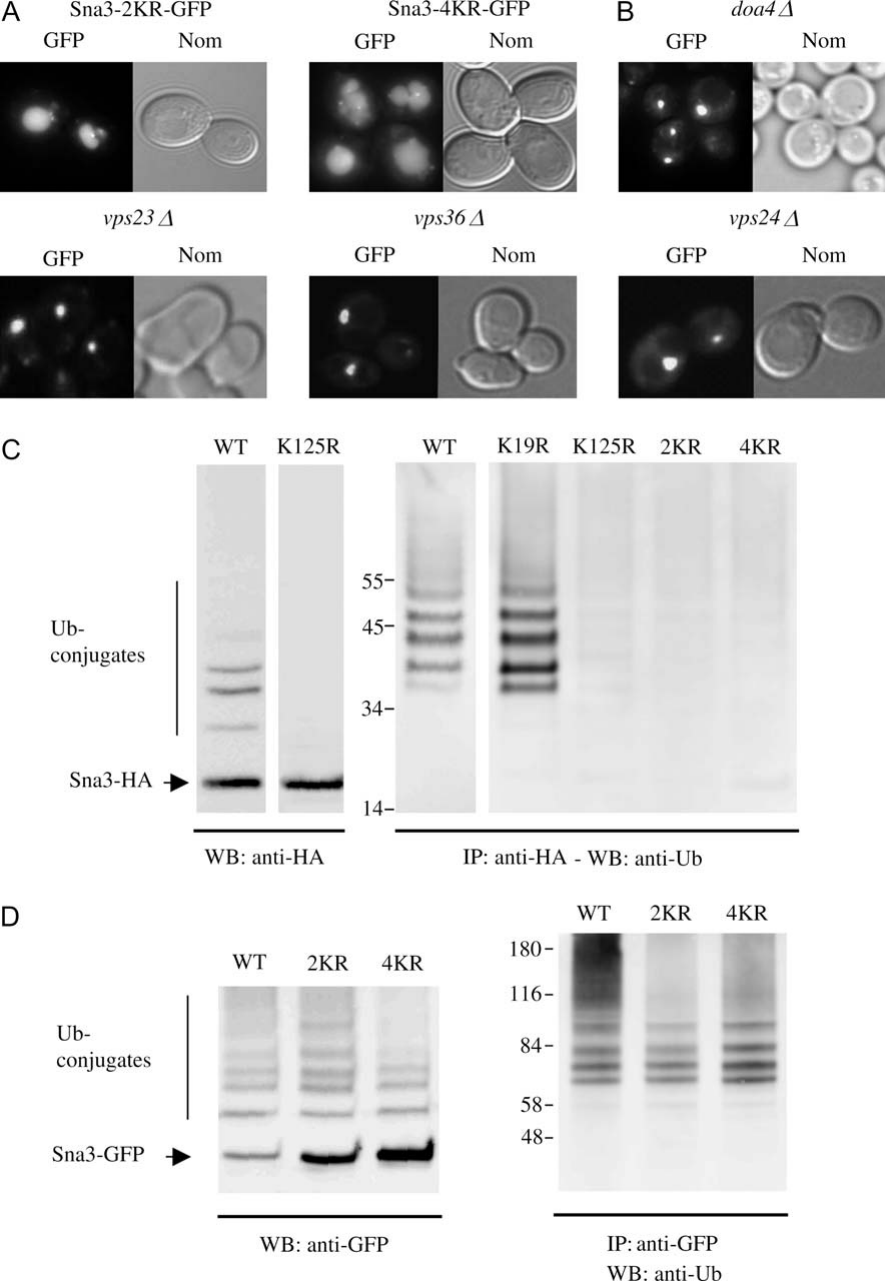
**Sna3p is polyubiquitylated on one target lysine, K125.**A) Cells expressing Sna3-K19,125R-GFP and Sna3-4KR-GFP were grown to mid-exponential growth phase, and cells were examined for fluorescence and with Nomarski (Nom) optics. B) *doa4Δ*, *vps23Δ*, *vps36Δ* and *vps24Δ* cells transformed by a Sna3-GFP encoding plasmid were grown to mid-exponential growth phase, and cells were examined for fluorescence and with Nomarski optics. C) Left panel: HA-tagged version of Sna3p and Sna3-K125R were detected by immunoblotting of corresponding cell lysates from wild-type (WT) cells with anti-HA antibodies. Right panel: Modified forms of Sna3-HA and its KR mutant derivatives were detected by immunoblotting with anti-Ub antibodies after immunoprecipitation with anti-HA antibodies. D) Modified forms of Sna3-GFP and its KR mutant derivatives were detected by immunoblotting with anti-GFP antibodies or by immunoblotting with anti-Ub antibodies after immunoprecipitation with anti-GFP. The sizes of molecular weight markers are indicated. IP: immunoprecipitation, WB: western-blot.

We expressed GFP-tagged Sna3p variants in which the four lysine residues of Sna3p (see Figure 1A) were replaced by Arg in various combinations, hence making ubiquitylation of these residues impossible, and we indeed observed a lumenal localization of Sna3-K19,125R–GFP and Sna3-4KR–GFP in cells otherwise deleted (Figure 4A) or not (data not shown) for the chromosomal copy of Sna3p. We thus eliminated the possibility that sorting of the mutant Sna3p occurs as a result of oligomerization with endogenous wild-type Sna3p. These data confirm the results obtained by Reggiori and Pelham (10). Sna3-GFP is targeted to the vacuolar lumen without being modified by Ub added to the Sna3 moiety of the chimeric protein. We further tested whether Sna3-GFP used all or part of the ESCRT machinery required for MVB sorting of ubiquitylated membrane cargoes. We expressed wild-type Sna3-GFP from the inducible *GAL* promoter in mutant cells deleted for each of the subunits of the ESCRT machinery. The Sna3-GFP fluorescence in *vps23Δ*/Tsg101 (ESCRT-I), *vps36Δ* (ESCRT-II) and *vp*s*24Δ* (ESCRT-III) mutant cells is shown in Figure 4B. Vps23p and Vps36p both possess a Ub-binding motif that recognizes ubiquitylated cargoes (3). In all cases, Sna3-GFP stained one or two big dots, adjacent to the vacuole, which might represent the class E compartment. Our data suggest that all the elements of the ESCRT machinery are required for Sna3-GFP sorting at the MVB. Strikingly, in our genetic background, in addition to the localization in the vacuolar lumen of some cells, a class E-type fluorescence was observed for Sna3-GFP expressed in *doa4Δ* cells (Figure 4B). A more systematic investigation of Sna3-GFP fate in *doa4Δ* cells from different genetic backgrounds showed more or less normal sorting in cells in early exponential phase, notably in the genetic background used by Reggiori and Pelham (10) with increasing defects at higher cell densities (i.e. late exponential growth phase) (data not shown), in agreement with the observation of decreased Ub content in *doa4Δ* cells along cell growth (31).

Two hypotheses could explain the ubiquitylation of the Sna3-KR–GFP variants. Sna3-GFP might be ubiquitylated on the Sna3p N-terminal residue, as reported in the case of certain proteins (32). Alternatively, the Sna3-KR–GFP variants might be ubiquitylated on the GFP tag as a result of their physical interaction with Rsp5p. This prompted us to analyze the Sna3p ubiquitylation pattern with the Sna3p variants tagged with HA, which does not carry any Lys residue. A Western blot of Sna3p-HA produced in wild-type cells displayed a pattern of several bands of lower mobility than that of the main Sna3p-HA band (Figure 4C). These low-mobility species form a ladder of Ub-conjugated bands with successive additions of 7.6 kDa to the main signal. In contrast, no band with lower mobility was observed for the variant Sna3-K125R-HA produced in wild-type cells. Immunoprecipitations were performed on the corresponding lysate with antibodies recognizing HA, and immunoprecipitated proteins were probed with anti-Ub antibodies. Probing with anti-Ub revealed a ladder of more than five bands corresponding to Sna3p-HA conjugated to Ub. Immunoprecipitations were also performed on lysates from HA-tagged versions of Sna3p variants in which each cytoplasmic Lys residue or combination of Lys residues was replaced by Arg (Figure 4C). An ubiquitylation pattern similar to that of the wild type was observed either directly on lysates or after immunoprecipitation followed by anti-Ub analysis in the case of variant Sna3-K19R-HA, whereas no Ub conjugates were observed when K125, K19,125 or all four lysines in Sna3p were changed to Arg. We conclude from these experiments that Sna3p is polyubiquitylated on one target lysine only, K125. In an identical set of experiments performed with GFP-tagged versions of the same Sna3-KR variants, we observed that the GFP-tagged proteins were still ubiquitylated when the two cytoplasmic lysines or even when all four Lys residues in Sna3p were replaced by Arg (Figure 4C). We reason therefore that Sna3-4KR–GFP is polyubiquitylated on the GFP part of the fusion protein. This modification thus most probably directs a correct sorting of Sna3-GFP into the MVB, even in the absence of ubiquitylation on Sna3p lysine residues.

### Sna3p ubiquitylation is required for its MVB sorting

**Figure 5:**
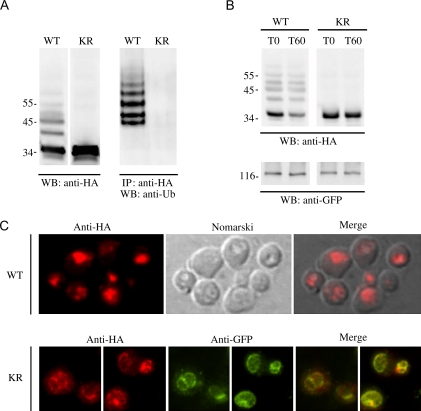
**Sna3p undergoes Ub-dependent MVB sorting.***sna3Δand VPH1-GFP* cells expressing Sna3p-6HA or Sna3p-4KR-6HA were grown to mid-exponential phase. A) Sna3p-6HA or Sna3p-4KR-6HA produced in *sna3Δ* cells were detected by immunoblotting of cell lysates with anti-HA antibodies (left panel) and immunoblotting with anti-Ub antibodies after immunoprecipitation with anti-HA antibodies (right panel). B) The production of Sna3p-6HA or Sna3p-4KR-6HA in *VPH1-GFP* cells was stopped by adding cycloheximide (100 μg/mL). Sna3p-6HA or Sna3p-4KR-6HA was detected by immunoblotting of cell lysates with anti-HA antibodies at the times indicated after the addition of cycloheximide, while Vph1-GFP was detected with anti-GFP antibodies. C) Phenylmethylsulfonyl fluoride was added for 1 h to*VPH1-GFP* cells producing Sna3p-6HA or Sna3p-4KR-6HA before the cells were fixed and processed for immunofluorescence with monoclonal anti-HA and polyclonal anti-GFP antibodies followed by Texas Red-labeled donkey anti-mouse IgG and FITC-labeled donkey anti-rabbit IgG as described in *Materials and Methods*. Cells were examined for fluorescence (the HA signal was detected using the rhodamine filter set and the GFP signal using the FITC filter set) and vacuole by Nomarski optics. IP: immunoprecipitation, WB: western-blot.

We investigated the role of Sna3p ubiquitylation in its MVB sorting both by biochemical and by immunofluorescence approaches using Sna3p tagged with HA, which does not carry any Lys residue. As immunofluorescent data using Sna3p-HA gave a signal that was too faint, we used a chimeric Sna3p carrying 6HA to improve the signal. We first checked the ubiquitylation fate of the fusion protein. Immunoblot of *Sna3Δ* cells expressing Sna3p-6HA revealed above the main Sna3p-6HA signal a ladder of bands of higher molecular weight, corresponding to ubiquitylated bands as evidenced by immunoprecipitation with antibodies against HA, followed by immunoblot with anti-Ub antibodies (Figure 5A). Sna3p-6HA ubiquitylation was completely abolished after mutation of four Sna3p lysines on the fusion protein, as revealed by both direct immunoblots and immunoprecipitation using anti-HA antibodies, followed by immunoblot with anti-Ub antibodies (Figure 5A). Hence, Sna3p-6HA mutated or not on its Lys residues was an appropriate tool for our purpose. We analyzed the stability and localization of Sna3-6HA and its four Lys-to-Arg counterpart in cells expressing a GFP-tagged version of Vph1p, a membrane subunit of the vacuolar adenosine triphosphatase (ATPase). We first investigated the turnover of Sna3-6HA and Sna3p-4KR-6HA after inhibiting protein synthesis by adding cycloheximide to exponentially growing cells (Figure 5B). Addition of cycloheximide for 1 h caused apparently no loss of Sna3p-4KR-6HA, while we observed a significant decrease in the amount of Sna3p-6HA compared with the control protein Vph1-GFP. This result indicates that a version of Sna3p that cannot be ubiquitylated is protected against vacuolar degradation. Next, we determined the respective localization of the two proteins by indirect immunofluorescence microscopy. To facilitate localization studies, phenylmethylsulfonyl fluoride (PMSF) was added for 1 h in exponentially grown cells to inhibit vacuolar proteases before cells were fixed and processed for immunofluorescence with anti-HA and anti-GFP antibodies (Figure 5C). Sna3p-6HA was clearly evidenced in vacuole interior, as judged by colocalization of the HA-specific fluorescent signal with vacuoles, detected by Nomarski optics. In contrast, we observed that the HA-specific signal of Sna3p-4KR-6HA co-localized with the GFP-specific signal of the vacuolar membrane marker, Vph1-GFP. Both biochemical and immunofluorescence microscopy data thus strongly suggest that the Lys-to-Arg version of Sna3p is not targeted to the vacuole interior. Therefore, as described for other MVB cargoes, Sna3p ubiquitylation is clearly required for its MVB sorting.

### Ubiquitin–Sna3p conjugates are extended through Ub Lys63

**Figure 6:**
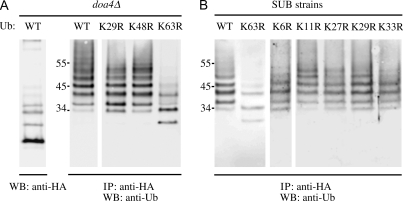
**Sna3p is modified by Lys63-linked Ub chains.**A) *doa4Δ* cells co-transformed with Sna3p-HA encoding plasmid and plasmids encoding wild-type (wt) Ub, UbK29R, UbK48R or UbK63R were grown to mid-exponential phase and induced for Ub synthesis for 1 h as described in Figure 3C. Modified forms of Sna3-HA and its KR mutant derivatives were detected by immunoblotting with anti-Ub antibodies after immunoprecipitation with anti-HA. The positions of size markers are indicated. B) Sna3-HA ubiquitylation in cells whose sole source of Ub was a modified Ub. SUB280 and derivative strains producing wild-type Ub or a modified form of Ub, respectively, were transformed Sna3p-HA encoding plasmid. Cells were grown to mid-exponential phase and modified forms of Sna3-HA were detected by immunoblotting with anti-Ub antibodies after immunoprecipitation with anti-HA. The sizes of molecular weight markers are indicated.

Little is as yet known about the ubiquitylation features associated with Golgi to vacuole trafficking. Some transporters misrouted from Golgi to vacuole are thought to undergo Rsp5-dependent polyubiquitylation, but the types of Ub chain have not been characterized (4,6,33). In contrast, MVB cargoes are thought to be primarily monoubiquitylated (30). We attempted to characterize ubiquitylation associated with Sna3p trafficking. Complementing Ub levels in *doa4Δ* cells with plasmid-encoded Ub provides a powerful tool for characterizing the types of Ub chain carried by target proteins (34,35). We therefore used *doa4Δ* cells to test the effect of various mutated Ubs on Sna3p-HA ubiquitylation. *doa4Δ* cells were grown to mid-exponential growth phase and the production of Ub variants was induced by adding copper salt for 1 h before harvest. By overexpressing wild-type Ub in *doa4Δ* cells, more than eight Ub conjugates were evidenced when Sna3p-HA was immunoprecipitated with anti-HA and Ub conjugates were revealed with anti-Ub antibodies (Figure 6A). The pattern of ubiquitylation was similar to that previously observed in wild-type cells (Figure 4C). Overexpression of UbK29R and UbK48R in *doa4Δ* cells also restored a normal ubiquitylation pattern of Sna3p-HA. In contrast, after overexpression of UbK63R, only mono- and di-ubiquitylated forms of Sna3p-HA were observed, together with some minor upper bands. These data demonstrated the inability of UbK63R to induce complete polyubiquitylation of Sna3p-HA. However, because Sna3p is ubiquitylated on a unique residue, Lys125, one would expect to observe only a monoubiquitylation of Sna3p-HA in cells overexpressing UbK63R. *doa4Δ* still produces some endogenous Ub. This wild-type Ub might compete for incorporation in Ub chains and could account for the presence of more than one added residue on Sna3p. In order to ascertain that the Ub profile observed upon overexpression of UbK63R did not result from the pool of chromosomal-encoded Ub, we investigated another experimental system. We analyzed the fate of Sna3p-HA in a strain in which the four natural Ub genes had been disrupted (36) and which expresses plasmid-encoded wild-type Ub or various Lys-to-Arg mutated Ub genes as their sole source of Ub (Figure 6B). The profiles of Sna3p–Ub conjugates were identical in cells producing wild-type Ub or mutated Ub with substitutions at Lys6, 11, 27, 29 and 33. The effect of substituting Lys48 was not tested because it is lethal for the cells (36). In contrast, the pattern of Ub–Sna3p conjugates in cells producing UbK63R as sole source of Ub was similar to that observed in *doa4Δ* cells overexpressing UbK63R, with the number of Ub conjugates notably reduced. The presence of more than one Ub-conjugated band still remains to be understood, but it is possible that when Lys63-Ub chains cannot be formed, an alternative chain is synthesized, at least in this case. Whatever the case, the overall data clearly indicate that Sna3p is modified by Lys63-Ub chains.

## Discussion

We investigated the role of Rsp5p and Ub in the intracellular sorting of Sna3p. While this paper was in revision, three reports also dealing on Sna3p trafficking were published (16–18). Some aspects of our data converge with those presented in these papers, but they also shed a novel and different light on the sorting of this particular MVB cargo, as detailed below. We show that Sna3p physically interacts with the E3 ligase Rsp5p and that this interaction is essential for sorting of Sna3p to the endosomal pathway. Sna3p is ubiquitylated by Rsp5p and modified by K63-linked Ub chains. In contrast to the conclusions from prior (10) or parallel (16–18) reports, we demonstrate that as reported for most other MVB cargoes, Sna3p ubiquitylation is required for its MVB sorting.

Sna3p–Rsp5p interaction was found to be dependent on the Sna3p PY motif and on the WW2/WW3 domains of Rsp5p. This interaction was demonstrated both *in vivo* by co-immunoprecipitation experiments and *in vitro* by pull-down experiments. This interaction was completely lost *in vivo* when Sna3p harbored point mutations in the PY motif or a deletion of this motif. However, some binding between Sna3-AY (or Sna3-Δ28, not shown) and Rsp5-WW2/WW3 could still be observed *in vitro*. This might result from binding of Rsp5p to another Sna3p signal because a Tyr15 signal is important for Sna3p trafficking (17). The binding of Sna3p to Rsp5p appeared to be an absolute requirement for the trafficking of Sna3p, in agreement with the parallel studies (16–18). In cells producing Sna3p variants with mutations in the PY motif that impair Rsp5p binding, Sna3-GFP was not targeted to the vacuolar lumen but remained in multiple mobile dots throughout the cytoplasm. The same pattern of Sna3p accumulation was also observed in *rsp5* mutant cells, characterized by a reduction in the level of Rsp5p (*npi1* mutant) or by temperature sensitivity (*rsp5-101*ts mutant) or by point mutations of conserved residues of the WW3 Rsp5p domain. An accumulation of Sna3-GFP in several dots was also reported in an *rsp5* catalytic mutant and in the case of a Sna3-PY mutant [(17); Figure 6]. The dots we observed have an appearance similar to that of vesicles in which Sna3-GFP accumulates in *pep12Δ* cells, deficient for fusion to the late endosome, suggesting that they might be of a similar nature. Thus, the Rsp5p–Sna3p interaction is crucial for Sna3p sorting to the endosomal pathway. The Sna3p sorting defect in *rsp5* mutants appears to differ from that observed for MVB cargoes such as Cps1 and Phm5. It was previously shown that GFP-Cps1p and GFP-Phm5p were missorted to the limiting membrane of the vacuole in *rsp5* mutant cells. These proteins were thus normally sorted from Golgi to endosomes but displayed deficient MVB sorting. In these cases, mutated Rsp5p causes insufficient Ub tagging of these biosynthetic cargoes for their correct entry into MVB pathway (7–9). Hence, it seems here that Rsp5p plays a novel and key role in Sna3p Golgi to endosome trafficking by virtue of its interaction with Sna3p, possibly prior to MVB sorting. Watson and Bonifacino also noted that impairing the interaction between Rsp5 and Sna3 led to its accumulation in a compartment that may lie upstream of MVBs (18). However, we cannot exclude on the basis of the our fluorescence data alone that a defective Rsp5p–Sna3p interaction triggers Sna3p Golgi to endosome sorting by an alternative route or leads to defective MVB sorting, followed by Sna3p recycling to some endosomes. The function fulfilled by Rsp5p in Sna3p sorting as a mere consequence of a strong interaction would be similar to the role played by the human homologue Nedd4 in Golgi to lysosome trafficking of the lysosomal-associated protein transmembrane 5 (LAPTM5) is sorted from the Golgi to lysosome by association of its PY motifs with Nedd4 WW domains (12).

The Rsp5p–Sna3p interaction and its resulting role in Sna3p sorting specifically required the WW3 domain of Rsp5p: Sna3p was correctly targeted in *rsp5*-*w1* and *rsp5-w2* but not in the *rsp5-w3* mutant, as also noted in Oestreich et al. (17). However, point mutations in each domain did not completely abolish Sna3p–Rsp5p interaction in co-immunoprecipitation experiments (data not shown), suggesting that the three WW domains might co-operate in the binding but that the WW3 domain displays a greater affinity. Our observation is in agreement with the description of the preference of Sna3p for the Rsp5p WW3 domain, reported in an attempt to identify the partners of the various yeast WW-domain-containing proteins by means of a protein microarray approach (37). Although all the various Rsp5p WW domains recognize PY motifs, they each have distinct partners and functions. For instance, GFP-Cps1p and GFP-Phm5p were recovered in the vacuolar membrane in both *rsp5-w2* and *rsp5-w3* mutants (7–9). This description of the Rsp5p WW3 domain’s involvement in trafficking of Sna3p adds yet another function to the list of those already identified for this domain.

One obvious consequence of interaction between Sna3p and Rsp5p is the ubiquitylation of Sna3p, and we demonstrate that Sna3-GFP ubiquitylation is indeed Rsp5 dependent, which is in agreement with the other reports (16–18). We show that in *rsp5* mutant cells (*npi1* or *rsp5ts*), the polyubiquitylated bands disappeared, and the prevalence of a monoubiquitylated species of Sna3p increased. This observation may suggest that an alternative E3(s) might be involved in Sna3p monoubiquitylation, at least when Rsp5 activity is deficient. However, we cannot exclude that the residual Rsp5p activity in these mutant cells might perform monoubiquitylation more easily than polyubiquitylation. Anyhow, Rsp5p would have the task of elongating Ub chains. The involvement of the Golgi-located E3 Tul1p involved in Cps1p sorting (28) seems unlikely because we observed normal Sna3p ubiquitylation and sorting in *tul1Δ* cells. In contrast, Cps1p was shown to be ubiquitylated by the concerted activity of the two E3 ligases Rsp5p and Tul1p, both required for correct trafficking (9). It should be noted that an Rsp5p-dependent polyubiquitylation is also important for Golgi to endosome trafficking of several plasma membrane proteins routed to the vacuolar protein sorting (VPS) pathway instead of the plasma membrane under certain nutrient/substrate conditions (4–6,33) or after misfolding (38).

Sna3p harbors four lysines, two of which have been predicted to be cytoplasmic (K19 and K125) (10). Systematic mutation of K125 alone, K19-K125 or the four Lys residues of Sna3p-HA and analysis of the ubiquitylation pattern of the resulting mutants enabled us to conclude that Sna3p-K125 is the unique target of Sna3p ubiquitylation, which is in agreement with the identification of Sna3p-K125 ubiquitylation in two different proteomic approaches (14,15). In wild-type cells, several Sna3p–Ub-conjugated bands were observed using HA-tagged Sna3p, indicating that Sna3p was obviously polyubiquitylated on this target Lys residue. We tried to characterize the type of ubiquitylation displayed by Sna3p using either *doa4Δ* mutant cells complemented by Ub variants or a family of strains unable to form one particular type of Ub chain. These experiments enabled us to conclude that the Sna3p-HA ubiquitylation pattern was modified only in cells that cannot build Ub chains extended by Ub Lys63, where the higher molecular weight species disappeared. Hence, Sna3p was modified on one target lysine by Ub chains linked by Ub Lys63. Rsp5-dependent ubiquitylation of Sna3p with Lys63-linked Ub chains is consistent with previous work showing that Rsp5p is more likely to form poly-Ub chains linked by the Lys63 of Ub *in vitro*(39) and *in vivo*(34,35,40,41). Our data are the first example of a yeast MVB cargo modified with Lys63-linked Ub chains. Ubiquitylation with Lys63-linked Ub chains may well also be a modification of MVB cargoes in mammals, as the endosome-associated deubiquitylating enzyme AMSH (associated molecule with the SH3 domain of STAM), which preferentially deubiquitylates Lys63-linked Ub chains *in vitro*(42), was recently described to be involved in deubiquitylation of an MVB cargo (43).

Like Cps1p and Phm5p, Sna3p follows the VPS pathway to the vacuole, in a process dependent successively on the endosomal t-SNARE Pep12p, the ESCRT complexes (present data) and the Vps4p ATPase [(10) and data not shown], which disassembles the last ESCRT complex (44). Hence, Sna3p MVB sorting depends on a machinery that has the task of recognizing ubiquitylated cargoes. Sna3p is ubiquitylated in wild-type cells, most probably as a consequence of interaction with Rsp5. It was previously claimed that Sna3p was normally sorted to the MVB after mutation of its two cytoplasmic Lys residues into Arg, i.e. when it was not ubiquitylated (10). In the reported experiments, Sna3p was fused to the GFP. We show in the present report that Sna3-GFP was ubiquitylated on the GFP moiety of the fusion protein. This observation strongly suggests that the sorting of Sna3-4KR-GFP to the vacuole interior results from the potential for ubiquitylation of the GFP tag. Indeed, we show that whereas Sna3p tagged with 6HA, and ubiquitylated, was correctly sorted to the MVB and vacuole interior, a mutant Sna3p-6HA devoid of target Lys residues, and not ubiquitylated, displayed deficient MVB sorting and was recovered at the vacuolar membrane. According to our data, therefore, Sna3p undergoes canonical Ub-dependent MVB sorting like the other yeast MVB cargoes. This interpretation differs from a conclusion reached in two parallel reports, in which the authors monitored the fate of Sna3-GFP and proposed that it undergoes Ub-independent MVB sorting (16,18). McNatt et al., however, did note the residual ubiquitylation displayed by Sna3-GFP after mutation of the cytoplasmic Lys of Sna3p moiety, or in the *rsp5* mutant they used, and did not completely exclude the idea that this might be sufficient to mediate Sna3-GFP MVB sorting. Similarly, Watson and Bonifacino insisted on the fact, that, in addition to Rsp5–Sna3p interaction, Rsp5p catalytic activity was required for proper Sna3-GFP MVB sorting.

In contrast to the ubiquitylation of Sna3-GFP on the GFP tag, it was previously shown that the GFP–Cps1 fusion protein with all lysines in Cps1p replaced by arginines was no longer ubiquitylated in an experiment in which the endogenous Cps1p was (30). This different behavior might be explained by the fact that Cps1p does not physically interact with Rsp5p, whereas Sna3-GFP does through its PY motif. It should also be noted that the extent of ubiquitylation of the GFP tag linked to Sna3p depends on the position of the tag in the chimeric protein. The C-terminal GFP used in most studies, including ours, appears to be especially accessible to ubiquitylation, while a chimeric GFP-Sna3 with an N-terminal GFP tag was less heavily ubiquitylated (17). These authors mutated all Lys of Sna3p in both GFP-Sna3 and Sna3-GFP. They observed that the rate of degradation of the various Sna3p chimeric proteins decreased (as follows: *Sna3-GFP* *>* *Sna3KR-GFP* *>* *GFP-Sna3KR*) proportionally to the extent of ubiquitylation. This observation is interesting and is in line with the observation that the rate of internalization of endocytic cargoes depends on the extent of ubiquitylation of these cargoes (33,45). In the report of Oestreich et al., Sna3p ubiquitylation was monitored in cells overexpressing HA-tagged Ub by immunoprecipitation with an anti-Sna3p antibody, followed by immunoblot with anti-HA antibody. These authors still observed MVB sorting of GFP-Sna3KR, although they did not note any ubiquitylation of this protein with their experimental approach. They concluded that Sna3p ubiquitylation was not required for MVB sorting but modulated the kinetics of delivery to MVB. They did not exclude, however, the possibility of a residual ubiquitylation undetectable in their analysis and, indeed, the cells contained endogenous untagged Ub in addition to HA-tagged Ub. Our data are simpler by far: Sna3p-6HA ubiquitylation was evidenced both by immunoprecipitation followed by Western blots and by direct immunoblots. Ubiquitylated bands disappeared after mutation of the target Lys, and there was a total correspondence between ubiquitylation and normal sorting, and absence of ubiquitylation and impaired sorting.

Like other MVB cargoes, Sna3p clearly undergoes Rsp5p-mediated ubiquitylation and MVB sorting, although with some distinct features. Intracellular Sna3p trafficking exhibits a novel dependence with regard to the Ub-protein ligase Rsp5p because Sna3p trafficking to the VPS pathway appears strictly dependent on its binding to Rsp5p. It is thus possible that Rsp5p acts both as a scaffold protein for the first Sna3p trafficking step and as an enzyme, required at least for proper Sna3p MVB sorting. A similar situation seems to prevail in the case of Nedd4-dependent LAPTM5 lysosomal sorting. In this case, Nedd4–LAPTM5 interaction is required for sorting from Golgi, but Nedd4-dependent LATPM5 ubiquitylation is dispensable for lysosome sorting (12). In contrast to the role played by Rsp5p in Sna3p Golgi to endosome sorting, defective Rsp5p-dependent ubiquitylation in *rsp5* mutant cells resulted in plasma membrane targeting instead of vacuolar targeting of some transporters (5,6) or correct sorting to the VPS pathway but impaired MVB sorting, and consequently accumulation in the vacuolar membrane instead of in the vacuolar lumen for other cargoes like Cps1p and Phm5p (7–9,33). None of the corresponding proteins displays a PY motif. They probably interact with Rsp5p via PY-containing adaptors such as Bul1/2p (5,6), Bsd2p (22) and Tre1/2p (46). The Bul1/2 proteins were the first to be identified as PY-containing Rsp5p partners. These two adaptors are involved in Rsp5p-dependent ubiquitylation of several plasma membrane Rsp5p substrates lacking PY motifs (4,5,38). Bsd2p and Tre1/2p act together in substrate-induced sorting of manganese Smf transporters to the VPS pathway instead of Golgi to plasma membrane targeting (22,46,47). Bsd2p and Tre1/2p are small membrane-bound proteins undergoing Rsp5 interaction and Rsp5p-dependent ubiquitylation and targeting to the vacuolar lumen. Impairment of the interaction of these three proteins with Rsp5p by mutation of their PY motives was described to result in impairment of Smf targeting (22,46), but whether the mutated proteins were also mislocalized was not investigated. Whether, similarly, Sna3p can act as an adaptor for Rsp5 substrates is currently under investigation.

## Materials and Methods

### Strains, plasmids, media and growth conditions

The *Saccharomycescerevisiae* strains used in this study are listed in Table 1. *npi2*/*doa4* is a specific allele of *DOA4* that carries a point mutation affecting a conserved residue, resulting in the same phenotype as complete *DOA4* deletion (35). Cells were transformed by the lithium method, as modified by Gietz et al. (48). Cells were grown at 30°C in YPD medium (1% yeast extract, 2% peptone, 2% glucose) or in YNB minimal medium containing 0.5% ammonium sulfate, 0.17% yeast nitrogen base (Difco, MI, USA), 2% glucose and supplemented with appropriate nutrients. 6His-tagged Ub was overproduced under the control of the *CUP1* promoter by growing the cells for 3 h in the presence of 0.1 mm CuSO_4_.

**Table 1:** Strains used in this study

Strain	Genotype	Source
27061b	*MAT***a***ura3 trp1*	(51)
27064b	*MAT***a***ura3 trp1 npi1*	(51)
JM07	*MAT***a***ura3 trp1 tul1::KanMX4*	(9)
MOB53	*MAT***a***ura3 trp1 doa4::kanMX4*	(33)
JM01	*MAT***a***ura3 trp1 npi1 doa4::kanMX4*	(9)
JM02	*MAT*α*ura3 trp1 npi2 tul1::kanMX4*	(9)
JM06	*MAT***a***ura3 trp1 npi1 tul1::kanMX4 doa4::kanMX4*	(9)
MOB54	*MAT***a***ura3 trp1 npi1 end3::KanMX4*	This study
BY4741	*MAT***a***his3Δ1 leu2Δ0 met15Δ0 ura3Δ0*	Euroscarf
BY4741 *VPH1-GFP*	*MAT***a***his3Δ1 leu2Δ0 met15Δ0 ura3Δ0 VPH1-GFP-HIS3MX6*	Invitrogen
BY4742*sna3Δ*	*MAT*α*his3Δ1 leu2Δ0 lys2Δ0 ura3Δ0 sna3::kanMX4*	Euroscarf
BY4742*pep12Δ*	*MAT*α*his3Δ1 leu2Δ0 lys2Δ0 ura3Δ0 pep12::kanMX4*	Euroscarf
BY4742*vps23Δ*	*MAT*α*his3Δ1 leu2Δ0 lys2Δ0 ura3Δ0 vps23::kanMX4*	Euroscarf
BY4742*vps36Δ*	*MAT*α*his3Δ1 leu2Δ0 lys2Δ0 ura3Δ0 vps36::kanMX4*	Euroscarf
BY4742*vps24Δ*	*MAT*α*his3Δ1 leu2Δ0 lys2Δ0 ura3Δ0 vps24::kanMX4*	Euroscarf
MK1	*MAT*α*his3-Δ200 leu2-3,112 ura3-52 lys2-801 trp1-1 RSP5::HA-RSP5*	(52)
BG1-1C	*SUP11 mod5-1 ade2-1 ura3 leu2-3,112 lys2-1 his4-519 rsp5::kanMX4* [YCplac111, *LEU2 3HA-RSP5*]	(27)
BG1-1C/w1	*SUP11 mod5-1 ade2-1 ura3 leu2-3,112 lys2-1 his4-519 rsp5::kanMX4* [YCplac111, *LEU2 3HA-rsp5-w1*]	(27)
BG1-1C/w2	*SUP11 mod5-1 ade2-1 ura3 leu2-3,112 lys2-1 his4-519 rsp5::kanMX4* [YCplac111, *LEU2 3HA-rsp5-w2*]	(27)
BG1-1C/w3	*SUP11 mod5-1 ade2-1 ura3 leu2-3,112 lys2-1 his4-519 rsp5::kanMX4* [YCplac111, LEU2 3HA-rsp5-w3]	(27)
SUB280	*MAT***a***lys2-801 leu2-3 112 ura3-52 his3-Δ200 trp1-1 ubi1-Δ1::TRP1, ubi2-Δ2:ura3, ubi3-Δub-2, ubi4-Δ2::LEU2* (pUB39 Ub, LYS2) (pUB100, HIS3)	(36)
SUB 413	*MAT***a***lys2-801, leu2-3,112, ura3-52, his3-Δ200, trp1-1(am) ubi1-Δ1::TRP1, ubi2-Δ2::ura3, ubi3-ΔUb-2, ubi4-Δ2::LEU2* (pUB39 UbK63R) (pUB100, HIS3)	(53)
SUB 515	*MAT***a***lys2-801, leu2-3,112, ura3-52, his3-Δ200, trp1-1(am) ubi1-Δ1::TRP1, ubi2-Δ2::ura3, ubi3-ΔUb-2, ubi4-Δ2::LEU2* (pUB39 UbK6R) (pUB100, HIS3)	Finley Laboratory
SUB 516	*MAT***a***lys2-801, leu2-3,112, ura3-52, his3-Δ200, trp1-1(am) ubi1-Δ1::TRP1, ubi2-Δ2::ura3, ubi3-ΔUb-2, ubi4-Δ2::LEU2* (pUB39 UbK11R) (pUB100, HIS3)	Finley Laboratory
SUB 517	*MAT***a***lys2-801, leu2-3,112, ura3-52, his3-Δ200, trp1-1(am) ubi1-Δ1::TRP1, ubi2-Δ2::ura3, ubi3-ΔUb-2, ubi4-Δ2::LEU2* (pUB39 UbK27R) (pUB100, HIS3)	Finley Laboratory
SUB 518	*MAT***a***lys2-801, leu2-3,112, ura3-52, his3-Δ200, trp1-1(am) ubi1-Δ1::TRP1, ubi2-Δ2::ura3, ubi3-ΔUb-2, ubi4-Δ2::LEU2* (pUB39 UbK29R) (pUB100, HIS3)	Finley Laboratory
SUB 519	*MAT***a***lys2-801, leu2-3,112, ura3-52, his3-Δ200, trp1-1(am) ubi1-Δ1::TRP1, ubi2-Δ2::ura3, ubi3-ΔUb-2, ubi4-Δ2::LEU2* (pUB39 UbK33R) (pUB100, HIS3)	Finley Laboratory

The plasmids used in this study are listed in Table 2. *Sna3* point mutations as well as nucleotide deletions were all generated by polymerase chain reaction (PCR) and cloned as *Hin*dIII–*Age*I fragments in the pTL321 vector creating a C-terminal GFP-tagged Sna3p under the control of a *TPI1* promoter (10). To construct *SNA3* under the control of *GAL1* inducible promoter, Sna3-GFP was cloned from pTL321 vector by PCR as a *Spe*I and *Xho*I fragment into the p415 vector (49). The Sna3-HA variants were constructed by PCR and cloned as *Hin*dIII and *Age*I fragments in the pTL321 vector creating C-terminal HA-tagged versions of Sna3p under the control of the *TPI1* promoter. Each construct was checked entirely by sequencing. All enzymes for manipulation of DNA and Faststart High Fidelity PCR polymerase were from Roche Applied Science, except *Age*I enzyme, which was from New England Biolabs.

**Table 2:** Plasmids used in this study

Plasmid	Genotype	Source
pRS416*-GFP-CPS1*	*CEN, URA3, TPI1-GFP-CPS1*	(10)
pRS416-*SNA3-GFP*	*CEN, URA3, TPI1-SNA3-GFP*	(10)
pRS416-*SNA3-HA*	*CEN, URA3, TPI1-SNA3-HA*	This study
pRS416-*SNA3-AY-GFP*	*CEN, URA3, TPI1-SNA3-AAAY-GFP*	This study
pRS416-*SNA3-Δ28-GFP*	*CEN, URA3, TPI1-SNA3-ΔCt28-GFP*	This study
pRS416-*SNA3-K2R-GFP*	*CEN, URA3, TPI1-SNA3-K19,125R-GFP*	This study
pRS416-*SNA3-4KR -GFP*	*CEN, URA3, TPI1-SNA3-4KR-GFP*	This study
P415-*SNA3-GFP*	*CEN, LEU2, GAL1-SNA3-GFP*	This study
pRS416-*SNA3-K19R-HA*	*CEN, URA3, TPI1-SNA3K-19R-HA*	This study
pRS416-*SNA3-K125R-HA*	*CEN, URA3, TPI1-SNA3K125R- HA*	This study
pRS416-*SNA3-2KR-HA*	*CEN, URA3, TPI1-SNA3-K19,125R- HA*	This study
pRS416-*SNA3-4KR -HA*	*CEN, URA3, TPI1-SNA3-4KR- HA*	This study
pRS416-*SNA3-6HA*	*CEN, URA3, TPI1-SNA3-6HA*	This study
pRS416-*SNA3-4KR-6HA*	*CEN, URA3, TPI1-SNA3-4KR- 6HA*	This study
pYEp96	*2*μ, *TRP1, CUP1-UBI*	(54)
pYEp96-6HIS-Ub	*2*μ, *TRP1*, CUP1–6His-UBI	This laboratory
pYEp96-K63R	*2*μ, *TRP1*, CUP1-UBI-K63R	(55)
pYEp96-K48R	*2*μ, *TRP1*, CUP1-UBI-K48R	(55)
pYEp96-K29R	*2*μ, *TRP1*, CUP1-UBI-K29R	(55)
pYEp96-7KR	*2*μ, *TRP1*, CUP1-UBI-7KR	(55)

### Protein extracts and western blotting

Total protein extracts were prepared as follows: cells were disrupted in a ‘One Shot’ Cell Disrupter (Constant Systems LDT, Daventry, UK) at maximum pressure of 2.7 10^8^ Pa. The disrupted cells were centrifuged twice (3000 × ***g*** for 3 min at 4°C) to remove unbroken cells. The resulting lysate was resuspended in dissociation buffer [final concentration: 4% sodium dodecyl sulfate; 0.1 m Tris–HCl, pH 8; 4 mm ethylenediaminetetraacetic acid (EDTA); 20% glycerol; 2% 2-mercaptoethanol and 0.02% bromophenol blue]. Proteins in dissociation buffer were heated at 95°C, resolved by SDS–PAGE in 10% acrylamide gels with tricine buffer and transferred to nitrocellulose membranes. The membranes were probed with monoclonal anti-GFP antiserum, or monoclonal anti-HA (clone 3F10) (Roche Diagnostics, Meylan, France), monoclonal anti-Ub (clone P4D1) and horseradish peroxidase conjugate (Santa Cruz Biotechnology, Santa Cruz, CA, USA), monoclonal anti-Nedd4 (BD Biosciences Europe, Erembodegen, Belgium) or polyclonal anti-3-phosphoglycerate kinase (PGK) (Molecular Probes Europe, Leiden, the Netherlands). Horseradish peroxidase-conjugated anti-mouse or anti-rabbit immunoglobulin G (IgG) was used as secondary antibodies (Sigma, St. Louis, MO, USA). Antibodies binding were detected by enhanced chemiluminescence.

### Immunoprecipitation

Cells were harvested by centrifugation at 4°C in the presence of 10 mm sodium azide and then washed once with cold water supplemented with 10 mm sodium azide and resuspended in 1.2 mL of cold lysis buffer (TNE buffer: 100 mm Tris–HCl, pH 7.5; 150 mm NaCl; 5 mm EDTA plus a mixture of protease inhibitors –‘Complete’ from Roche diagnostics – and 25 mm freshly prepared *N*-ethylmaleimide). They were then disrupted in a ‘One Shot’ Cell Disrupter (Constant Systems LDT). The disrupted cells were centrifuged twice (3000 × ***g*** for 3 min at 4°C) to remove unbroken cells, and the resulting lysate was subjected to centrifugation at 13 000 × ***g*** for 1 h at 4°C to generate the supernatant and pellet fractions. The pellet was resuspended in 400 μL of lysis buffer and 100 μL of 50% trichloroacetic acid was added. After 30 min of incubation on ice, cells were collected by centrifugation (10 min, 13 000 × ***g***). They were washed with 0.6 mL Tris Base 1 m (without resuspending the pellet) and centrifuged for 15 seconds. The pellet was resuspended in 60 μL of sample buffer without 2-mercaptoethanol and incubated for 10 min at 95°C. Then, 0.6 mL of TNET (TNE buffer supplemented with 1% Triton-X-100) buffer was added, and the mixture was centrifuged at 12 000 × ***g*** for 25 min at 4°C. The supernatant was recovered and the antibodies (10 μL of anti-GFP or 10 μL anti-HA) were added. The tubes were incubated on ice for 1 h and then 50 μL of freshly prepared protein G–Sepharose beads (Gamma Bind G Sepharose; Amersham Pharmacia, Biotech AB) was added and incubated with gentle rocking at 4°C overnight. The next day, the protein G beads with bound proteins were pelleted by centrifugation (3000 × ***g***, 1 min) and washed four times with TNET buffer. The pellet was resuspended in sample buffer, incubated for 10 min at room temperature and then heated for 10 min at 95°C before resolution by SDS–PAGE and analysis by immunoblotting.

For the co-immunoprecipitation experiment, cells were resuspended in buffer A (250 mm sorbitol, 10 mm imidazole, 10 mm EDTA, 1 mm PMSF and 1 g/mL protease inhibitor mixture, pH 7.5). After disruption, the cleared cell lysate was centrifuged at 20 000 × ***g*** for 45 min at 4°C. The pellet was resuspended in buffer B (10 mm imidazole and 10 mm EDTA, pH 7.5). Crude membranes were solubilized by 0.5% lysophosphatidylcholine (LPC) for 1.5 h at 4°C with a rotating movement. The unsolubilized membranes were removed by centrifugation at 100 000 × ***g*** for 1 h at 4°C. Using rat anti-HA or mouse anti-GFP antibodies and protein G beads, we performed immunoprecipitation for 2–4 h at 4°C with a rocking movement. The beads were washed twice with buffer C [10 mm imidazole, 10 mm EDTA, 150 mm NaCl and 1% Nonidet P-40 (NP-40), pH 7.5], once with buffer D (10 mm imidazole, 10 mm EDTA, 250 mm NaCl and 0.1% NP-40, pH 7.5) and immunoprecipitated proteins were eluted with sample buffer.

### GST pull-down

*Escherichia coli* BL21 expressing GST or GST-WW2/WW3 of Rsp5p from the pGEX4T-1 plasmid (21) were grown at 37°C upto A600 = 0.6. Protein expression was induced overnight at 18°C with 200 μg/mL of Isopropyl-β-D thiogalactopyranoside (IPTG). Fusion proteins were purified on glutathione–Sepharose 4B beads (Amersham Biosciences, Uppsala, Sweden) according to the manufacturer’s instructions. Yeast cells transformed with plasmids pRS416-*TPI1-SNA3-GFP* or pRS416-*TPI1-GFP-CPS1* were incubated overnight to exponential growth phase in liquid synthetic medium. Cells in the exponential growth phase were harvested, and the pellet was resuspended in 1.2 mL lysis buffer [50 mm Tris, pH 7.4, 150 mm NaCl, 10 mm MgCl_2_, 0.1% Triton-X-100, 1 mm dithiothreitol (DTT), 10% glycerol plus protease inhibitors (‘Complete EDTA-free’; Roche Diagnostics, Meylan, France) and 25 mm*N*-ethylmaleimide], and cell lysate was prepared as described. The pull-down reaction was performed in the final volume of 1 mL with 200 μg GST or GST-WW2/WW3 proteins bound to 100 μL of beads and the cell lysate corresponding to 2 × 10^8^ in lysis buffer. The reaction was incubated for 1 h at 4°C with gentle rocking and then centrifuged twice (1 min, 2000 ***g*** at 4°C). An aliquot of the supernatant corresponding to the unbound fraction (NB) was taken and heated at 95°C for 5 min in the presence of sample buffer. The beads were washed five times with 1 mL of lysis buffer and then the proteins that were bound (B) to the beads were eluted with 50 μL dissociation buffer and heated for 10 min at 95°C. Proteins in dissociation buffer were resolved by SDS–PAGE in acrylamide gels and probed with anti-GFP antiserum to detect Sna3p or Cps1p and anti-HA antiserum to detect Bul1p.

### 6His-tagged ubiquitylated protein purification

6His-tagged ubiquitylated protein purification experiments were performed as previously described (9). *doa4Δ* cells producing Sna3-GFP and 6His-Ub under the control of the *CUP1* promoter were grown overnight in selective medium to mid-exponential growth phase, then incubated with 0.1 mm CuSO_4_ for 3 h. 6 × 10^8^ to 7 × 10^8^ cells were harvested by centrifugation at 4°C in the presence of 10 mm sodium azide. The cells were washed once and resuspended in 1.2 mL of cold lysis buffer (50 mm Tris–HCl, pH 7.4; 150 mm NaCl plus a mixture of EDTA-free protease inhibitors – Complete from Roche diagnostics – and 25 mm freshly prepared *N*-ethylmaleimide to prevent artifactual deubiquitylation). They were then disrupted in a ‘One Shot’ Cell Disrupter (Constant Systems LDT) at a maximum pressure of 2.7 kbar. The disrupted cells were centrifuged twice (3000 × ***g*** for 3 min at 4°C) to remove unbroken cells, and the resulting lysate was subjected to centrifugation at 13 000 × ***g*** for 30 min to generate the supernatant and pellet fractions. The pellet was resuspended in 300 μL of buffer A (lysis buffer supplemented with 5 mm imidazole, 0.1% sodium dodecyl sulfate and 1% Triton-X-100). The suspension was incubated on ice for 30 min, then diluted by adding 300 μL of buffer B (lysis buffer supplemented with 5 mm imidazole and 1% Triton-X-100) and centrifuged for 10 min at 13 000 × ***g*** to remove the remaining nonsoluble material. The supernatant was then passed through 200 μL Ni-NTA Superflow resin (Qiagen Inc., Hilden, Germany) packed into a disposable polypropylene column (Mini Bio-Spin chromatography columns; Bio-Rad). The unbound fraction was collected, and the resin was washed three times with 200 μL of buffer B. The 6His-tagged ubiquitylated proteins were eluted by three passages of 200 μL of elution buffer (50 mm Tris–HCl, pH 7.4; 150 mm NaCl; 200 mm imidazole) through the column. At each step, a 5-μL aliquot was withdrawn for Western blot analysis.

### Fluorescence microscopy and vacuole staining

Cells grown to exponential growth phase in YNB medium were supplemented with 5 μm Cell Tracker Blue CMAC (10 mm stock solution in dimethyl sulfoxide; Molecular Probes, Eugene, OR, USA) and incubated for 15 min at 30°C. They were then washed with YNB medium and concentrated by a factor of 10 by centrifugation. Cells were viewed immediately, without fixation, under a fluorescence microscope (type BY61; Olympus, Tokyo, Japan) and images captured with a digital camera. The results presented are based on our observations of >50 cells.

### Immunofluorescence

Yeast cells grown to exponential growth phase were incubated with 200 mm PMSF at 30°C for 1 h to inhibit vacuolar proteases (50). Next, cells were fixed by incubation for 45 min in 3.7% formaldehyde in buffer A (1.2 m sorbitol, 20 mm potassium phosphate, pH 7.4). They were then collected by centrifugation and resuspended for 8 min in buffer B (10 mm DTT, 0.1 m Tris–HCl, pH 9.4). They were washed twice in buffer A, resuspended in 2 mL of buffer A supplemented with 0.2 mg/mL Zymolyase 20 T (Seikagaku, Tokyo, Japan) and incubated for 15–30 min at room temperature. The resulting spheroplasts were washed twice in buffer A and were spotted onto polylysine-coated slides for 5 min. They were then permeabilized by incubation in PBS (50 mm potassium phosphate, pH 7.5 and 150 mm NaCl) supplemented with 0.1% Triton-X-100 for 2 min on ice (to visualize the HA epitope in the vacuolar interior) or for 10 min (to visualize the HA and GFP epitopes at the vacuolar membrane). The slides were rinsed with PBS and saturated in PBS plus 2% BSA for 30 min at room temperature. The slides were then incubated overnight at 4°C with the monoclonal anti-HA diluted 1:100 (Eurogentec, Liege-4102 Seraing-Belgium) or 1:1000 (polyclonal anti-GFP) in PBS supplemented with 0.2% BSA. They were then washed three times in PBS and incubated with Texas Red-conjugated donkey anti-mouse IgG and fluorescein isothiocyanate (FITC)-donkey anti-rabbit IgG (Jackson ImmunoResearch Laboratories, West Grove, PA, USA) diluted 1:100 for 30 min. The slides were mounted in Citifluor (Citifluor, London, UK). Samples were viewed under a microscope (type BY61; Olympus) with FITC and rhodamine filter sets and Nomarski optics.
